# FDG-PET/CT in the Radiotherapy Treatment Planning of Locally Advanced Anal Cancer: A Monoinstitutional Experience

**DOI:** 10.3389/fonc.2021.655322

**Published:** 2021-07-01

**Authors:** Clelia Di Carlo, Maika di Benedetto, Lisa Vicenzi, Sara Costantini, Francesca Cucciarelli, Francesco Fenu, Eleonora Arena, Cristina Mariucci, Maria Montisci, Valeria Panni, Fabiola Patani, Marco Valenti, Andrea Palucci, Luca Burroni, Giovanna Mantello

**Affiliations:** ^1^ Department of Radiation Oncology, Ospedali Riuniti Umberto I°, GM Lancisi, G Salesi, Ancona, Italy; ^2^ Department of Medical Physics, Ospedali Riuniti Umberto I°, GM Lancisi, G Salesi, Ancona, Italy; ^3^ Department of Nuclear Medicine, Ospedali Riuniti Umberto I°, GM Lancisi, G Salesi, Ancona, Italy

**Keywords:** anal cancer, 18FDG PET/CT, radiotherapy planning, dose escalation, target volume definition

## Abstract

**Aims:**

Radiotherapy with concurrent 5-fluorouracil/mitomycin-C based chemotherapy has been established as definitive standard therapy approach for anal cancer. Intensity Modulated Radiotherapy (IMRT) leads to a precise treatment of the tumor, allowing dose escalation on Gross Tumor Volume (GTV), with a surrounding healthy tissues sparing. Our study assessed the impact of 18-Fluorodeoxyglucose positron emission tomography (18FDG-PET/CT) on the radiotherapy contouring process and its contribution to lymphatic spread detection, resulting to a personalization of Clinical Target Volume (CTV) and dose prescription.

**Methods:**

Thirty-seven patients, with histologically proven squamous cell carcinoma of the anal canal (SCCAC) were analyzed. All patients were evaluated with history and physical examination, trans-anal endoscopic ultrasound, pelvis magnetic resonance imaging (MRI), computed tomography (CT) scans of the chest, abdomen and pelvis and planning 18FDG-PET/CT. The GTV and CTV were drawn on CT, MRI and 18FDG-PET/CT fused images.

**Results:**

Thirty-four (91%) out of 37 patients presented lymph nodes involvement, in one or more areas, detected on 18FDG-PET/CT and/or MRI. The 18FDG-PET/CT showed positive lymph nodes not detected on MRI imaging (PET+, MRI−) in 14/37 patients (38%). In 14 cases, 18FDG-PET/CT allowed to a dose escalation in the involved nodes. The 18FDG-PET/CT fused images led to change the stage in 5/37(14%) cases: four cases from N0 to N1 (inguinal lymph nodes) and in one case from M0 to M1 (common iliac lymph nodes).

**Conclusions:**

The 18FDG-PET/CT has a potentially relevant impact in staging and target volume delineation/definition in patients affected by anal cancer. In our experience, clinical stage variation occurred in 14% of cases. More investigations are needed to define the role of 18FDG-PET/CT in the target volume delineation of anal cancer.

## Introduction

Anal cancer is a rare disease accounting for 1–2% of digestive tract tumor in Europe and it is strongly related to HPV infection in as many as 90% of cases.

At diagnosis, 50% of anal cancer results confined to the primary site, 30% presents regional lymph nodes (LN) involvement while distant metastases are reelevated in less than 10% of cases (1, 2).

In locally advanced stage, the standard of care is represented by concurrent radio-chemotherapy with 5-fluorouracil and mitomycin C. Surgical resection is an option for non-responders or recurrent disease (3).

For locoregional staging, magnetic resonance imaging (MRI) represents the gold standard in detecting tumor extension and the involvement of adjacent structures such as muscles and soft tissues. Thorax and abdomen computed tomography (CT) is used to assess distant extent of spread, mainly metastasis in liver and lungs. In recent years, 18-Fluorodeoxyglucose (18FDG) positron emission tomography (PET/CT) is having an increasing role in staging and treatment planning of anal carcinoma, because of the high 18FDG-PET/CT avidity of this tumor (4–6).

In a recent meta-analysis of 17 studies, 18FDG-PET/CT showed a sensitivity of 99% in the detection of primary tumor compared to 67% of contrast enhanced CT. Moreover, 18FDG-PET/CT had an overall sensitivity of 93% and specificity of 76% for inguinal LN identification (7).

Currently, 18FDG-PET/CT is not part of routine staging in anal cancer and does not replace diagnostic CT. Nevertheless, several studies have reported the usefulness of pre-treatment 18FDG-PET/CT to better identify the extension of disease as well as to define the clinical volumes for radiation therapy (1–8).

The aim of the present study is to analyze the potential impact of 18FDG-PET/CT in the staging and target volume delineation of patients affected by anal cancer candidate to curative radio-chemotherapy.

## Methods

We retrospectively analyzed 37 patients with histologically proven squamous cell carcinoma of the anal canal (SCCAC), treated in our Institute between May 2012 and September 2020.

### Inclusion Criteria

From the total of anal cancer patients treated in our Institute, we selected patients with SCCAC that performed, for clinical staging, trans-anal endoscopic ultrasound, pelvis MRI, total body CT scans with contrast enhancement and planning 18FDG/PET-CT. We included patients with stages I–IV disease.

### External Beam Radiotherapy

For radiotherapy treatment, simulation was performed in supine position with a head rest and knee fixation. A planning CT was acquired from the diaphragm to the proximal diaphysis of the femur, with a slice thickness of 2 mm and adequate bladder filling.

### The 18FDG-PET/CT Acquisition

Within one week from planning CT, all patients underwent planning 18FDG-PET/CT in the Nuclear Medicine Department in treatment position using a GE Discovery 690 PET/CT scanner with a multi-detector-row CT component. Patients were fasted for at least 6 h prior to scanning. Blood glucose was assessed before starting the diagnostic investigation. The value of 200 mg/dl was identified as the upper limit of glucose blood level allowed to proceed with the scan. PET/CT images were acquired 60 ± 5 min after an intravenous injection of 18FDG. The dose was administered based on patient’s weight (3.0 MBq/kg). The PET-CT scan was performed in caudal-cranial direction with 3 min acquisition time per bed position from the base of skull to the middle of the thigh. All images were evaluated by two medical experts in nuclear medicine. PET images analysis was conducted based on qualitative and semi-quantitative analysis. Maximal standardized uptake value (SUVmax) was obtained drawing a Volumes of Interest (VOI) on any suspected pathological LN detected.

Moreover, the MRI imaging and the 18FDG-PET/CT were reviewed by an expert radiologist and expert nuclear medicine physician during multidisciplinary discussions.

### Radiotherapy Volume Delineation and Planning

Pelvis MRI, contrast enhanced CT and planning 18FDG-PET/CT were fused with radiotherapy planning CT using automatic co-registration and manually corrected when necessary.

The primary clinical target volume (CTV T) was delineated including gross tumor volume (GTV T), the entire anal canal and sphincter muscles as recommended by contouring atlas guidelines (9–11). Ten millimeters were added to CTV T to obtain the planning target volume (PTV T).

The nodal CTV (CTV N) included the mesorectum (delineated separately and expanded of 10 mm to define the internal margin), internal, external, presacral, obturator and inguinal LN areas and ischiorectal fossa. Common iliac LN was included only when involved. Seven millimeters margin were added to CTV N to generate PTV N including mesorectal expansion.

The MRI and/or 18FDG-PET/CT positive LN were delineated as GTV N and expanded of 5 mm to generate PTV.

### External Beam Radiotherapy

A total dose of 45 Gy (1.8 Gy/die) was delivered to CTV N with a simultaneous integrated boost (SIB) up to 54 Gy (2.16 Gy/die, EQD2[α/β10] = 54.7Gy) to CTV T. When indicated, an additional sequential boost on residual disease (GTV T) up to 59 Gy was prescribed. Moreover, on pathological LN (GTV N), we prescribed a SIB with total dose of 50–54 Gy (2–2.16 Gy/die, EQD2[α/β10] = 54.7Gy).

Intensity Modulated Radiotherapy (IMRT) treatment planning was elaborated for all patients. According to our Institute protocol, daily image guided radiotherapy (IGRT) with cone-beam CT (CBCT) was performed before treatment.

### Chemotherapy

All patients received concomitant chemotherapy with 5-fluorouracil (5FU)/mitomycin-C as recommended by Italian Association of Radiation Oncology (AIRO) and European guidelines: intravenous continuous infusion of 5-FU 1,000 mg/m^2^/day, days 1–4 and 29–32; Mitomycin 10 mg/m^2^, bolus days 1 and 29 (1).

## Results

A total of 37 patients with SCCAC were included in the study, 28 (76%) female and nine (24%) male. Median age was 55 years (range 40–88). Patients’ characteristics and clinical stage are reported in **Table 1**.

**Table 1:** Patient and tumor characteristics.

Total number	37
Gender	28 F
	9 M
Median age	55 (range 40–88)
Performance status	30 ECOG0
	7 ECOG1
Histology	37 SCC
Clinical stage (NCCN 2020)	1 T1N0M0 I
	1 T1N1M0 IIIA
	6 T2N0M0 IIA
	6 T2N1M0 IIIA
	1 T3N0M0 IIB
	10 T3N1M0 IIIC
	10 T4N1M0 IIIC
	1 T2N1M1 IV
	1 T3N1M1 IV

The median follow-up was 19 months (range 2–62). At the last follow-up, according to Response Evaluation Criteria In Solid Tumors (RECIST), 33 (89%) patients had a disease remission while three (8%) patients and one (3%) patient had local and systemic progression of disease, respectively.

**Figure 1:**
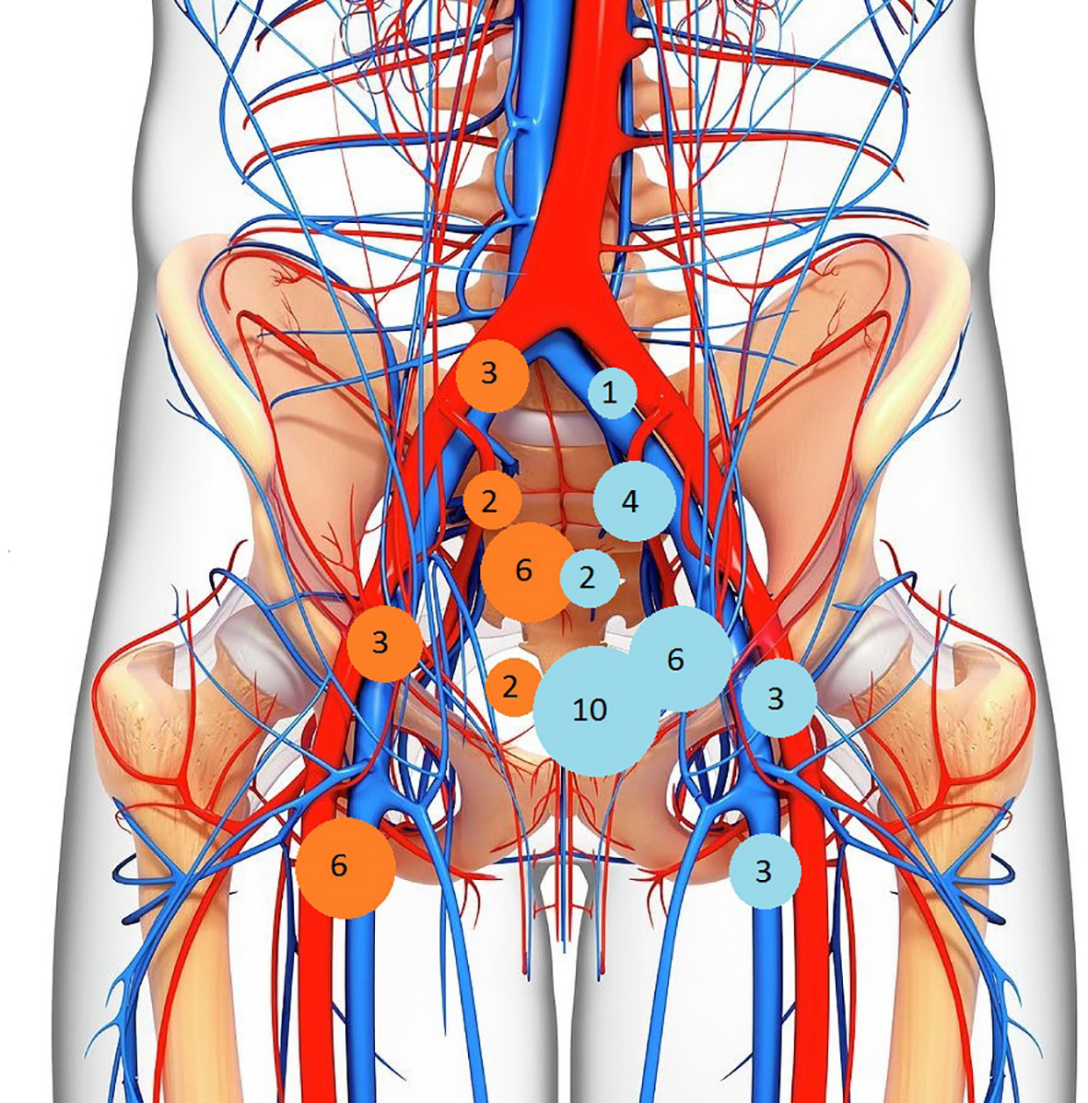
Mapping of cases with PET-positive (orange) and MRI-positive (light-blue) lymph nodes.

In 34/37 (91%) patients there was lymph nodal involvement detected on 18FDG-PET/CT and/or MRI. The 18FDG-PET/CT showed positive LN not detected on MRI imaging in 14/37 (38%) patients: two cases in common iliac LN, two in the internal iliac LN area, three in the external LN area, seven in the inguinal LN area, six in the presacral area and two in the mesorectal space. In 5/37 (14%) cases there was complete accordance between 18FDG-PET/CT and MRI in detecting LN involvement (**Table 2**). A mapping of number of patients with involved LN was carried out, mainly comparing MRI and 18FDG-PET/CT results (**Figure 1**).

**Table 2:** Distribution of positive lymph-nodes in our sample of patients detected with 18FDG-PET/CT and/or MRI.

IMAGING	LYMPH NODES (number of cases)						
	INGUINAL	COMMON ILIAC	INTERNAL ILIAC	OBTURATOR	EXTERNAL ILIAC	PRESACRAL	MESORECTAL
MRI− PET+	6	3	2	0	3	6	2
MRI+ PET+	13	2	0	0	1	3	8
MRI+ PET-	3	1	4	6	3	2	10

**Figure 2:**
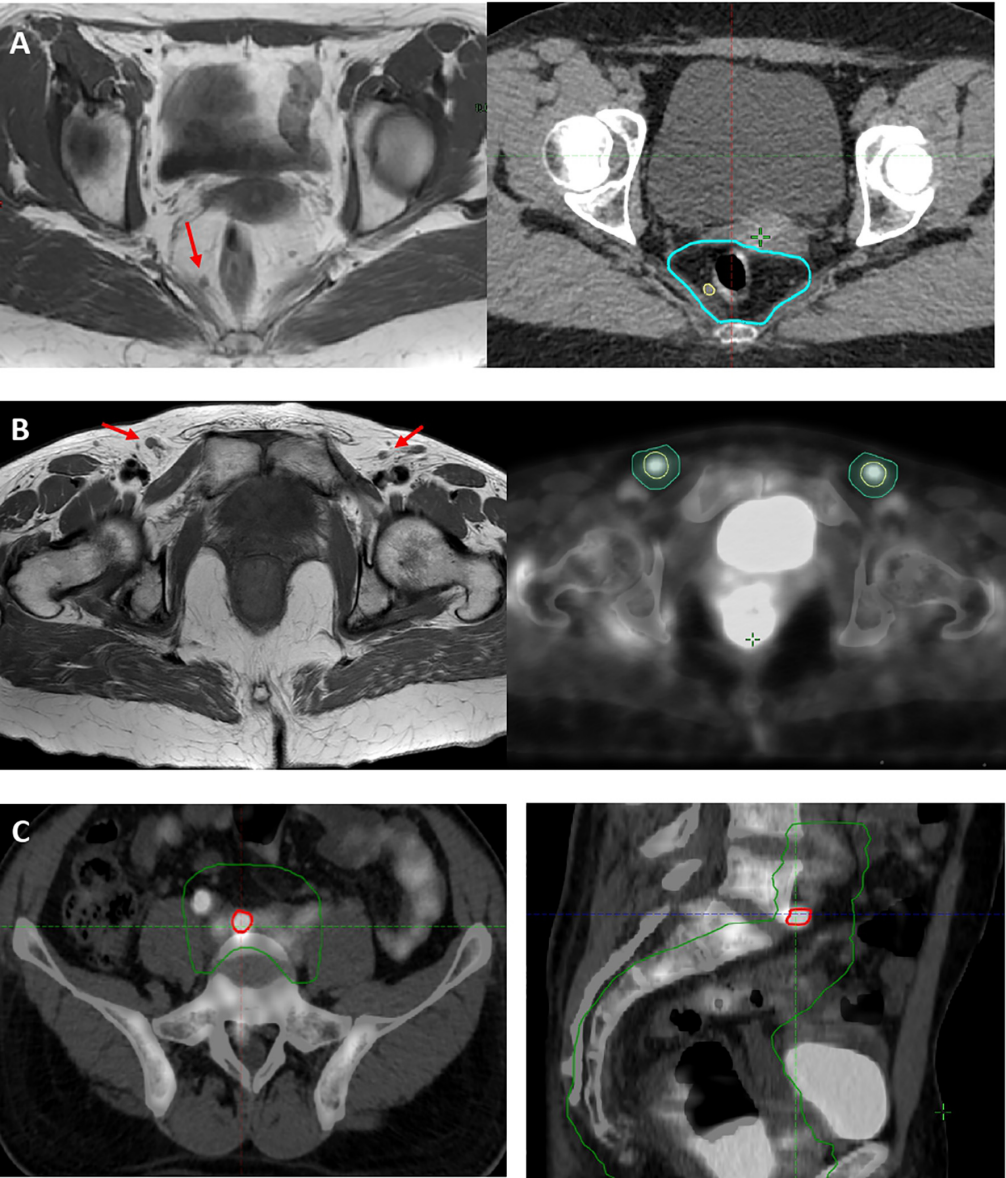
**(A)** Case of mesorectal PET−/MRI+ LN (red arrow), included as mesorectal GTV N (yellow line) in 54 Gy high-dose volume (light blue line). **(B)** Case of disease upstage and dose escalation on MRI− (red arrows)/PET+ (yellow line) inguinal LN. **(C)** Case of disease upstage with a dose escalation on MRI−/PET+ common iliac LN (red line) and inclusion of common iliac LN level to 45 Gy low-dose volume (green line).

The 18FDG-PET/CT planning led to change the stage in five (14%) cases when compared to MRI, particularly in four (11%) cases the stage changed from N0 to N1 for positive inguinal LN and in one (2.5%) case from M0 to M1 for common iliac LN involvement. In 10/20 (50%) patients with positive mesorectal LN, MRI outperformed 18FDG-PET/CT in detecting LN in this area. The 18FDG-PET/CT helped us to target volume delineation: in one case with PET-positive common iliac LN, the CTV was extended cranially to include this area (**Figure 2**). In 14/37 (38%) patients 18FDG-PET/CT led to a dose escalation on PET-positive LN reaching 50–54 Gy.

## Discussion

In clinical staging of anal cancer, MRI represents the gold standard to detect the tumor lesion, the involved adjacent anatomical structures and the adjacent loco-regional lymphatic spread. The abdominal and pelvic lymphadenopathies and distant metastasis are usually assessed with contrast enhanced CT. In the recent years the role of 18FDG-PET/CT in the staging of SCCAC has been growing probably due to high FDG-avidity of SCCAC as reported in the literature.

According to the NCCN guidelines, 18FDG-PET/CT may be considered to verify anal canal cancer staging especially to evaluate pelvic LN with normal size on CT imaging. Similarly, in the ESMO guidelines 18FDG-PET/CT is considered optional but is often recommended (1).

A recent meta-analysis of 17 studies compared the role of PET/CT with conventional imaging in the staging, response evaluation and follow up of patients with anal canal cancer. The authors calculated the pooled sensitivity and specificity for detection of LN involvement by 18FDG-PET/CT at 93 and 76%, respectively (7). Moreover, several studies have shown that 18FDG-PET/CT led to upstaging in about 20% of cases changing TNM stage in 21% and altering treatment strategy in 3–5% of cases (1, 12–16).

The use of 18FDG-PET/CT may also impact on radiotherapy planning as shown in three systematic reviews and meta-analyses where treatment planning was modified from 12 to 59% of patients based on PET/CT results (7, 17–19).

The present study, similarly to Krengli et al., aims to analyze the potential impact of 18FDG-PET/CT on tumor staging and treatment strategy in the management of SCCAC and investigates how 18FDG-PET/CT changed volume delineation in the radiotherapy treatment planning (20).

According to the literature, we found that MRI resulted more sensitive in T staging and provided more details to local extension, remaining the modality of choice for primary GTV contouring (21).

On the other hand, in our study we observed that 18FDG-PET/CT had a higher implication on nodal staging, particularly on regard to inguinal LN, leading to upstaging in the 14% of cases: in four (11%) from N0 to N1 for positive inguinal LN and in one (3%) from M0 to M1 for common iliac LN involvement.

Similar results were obtained in a study by Zimmerman et al. that evaluated 26 patients and reported about 13% of upstaging. Analogous results are showed in a recent meta-analysis by Jones and colleagues, with upstaging rate of 15%. Mahmud systematically review the literature to investigate the utility of 18FDG-PET/CT in the clinical staging and found that PET/CT identified distant metastatic sites not seen on conventional imaging in 2.4 to 4.7% of cases in agreement with our data (2.5%). No case of downstaging was reported in our series contrary to the literature where the use of 18FDG-PET/CT led to about 15% of downstaging (7, 21).

The influence of 18FDG-PET/CT findings on target volume definition and treatment planning is quite variable. Bhuva et a reported a series of 43 patients undergone 18FDG-PET/CT in addition to routine CT and MRI (8). The 18FDG-PET/CT imaging altered nodal stage in 32% of cases; however, despite these findings, all treatment plannings were not modified. In the study by de Winton and colleagues, 18FDG-PET/CT changed the management in 16% (10/61) of cases. Particularly, the addition of 18FDG-PET/CT to clinical staging had a considerable impact on treatment intent in 3% of patients and changed radiotherapy fields or technique, including or not nodal disease, in 13% of cases (18). Nguyen et al. analyzed 50 patients with SCCAC where pre-treatment 18FDG-PET/CT identified additional involved nodal groups causing radiotherapy treatment planning amendments in 19% (19).

In our sample, 18FDG-PET/CT allowed a dose escalation on PET positive LN in 14 (38%) patients using SIB.

Krengli et al. reported that 18FDG-PET/CT changed GTV and CTV contours in 55 and 37% of cases, respectively, with high rate of local control and low rate of late toxicity (20). Drapper et al. carried out a retrospective study of thirty-seven patients and compared three different contouring guidelines for pelvic LN. They showed how 18FDG-PET/CT imaging changed the contouring of LN areas and that LN “misses” generally appeared cranially (common iliac or para-aortic) or caudally (inguinal) to the recommended CTVs (22).

A recent study by Fiorentino et al., analyzed the role of 18FDG-PET/CT for the radiotherapy planning definition of the biological target volume in several pathologies including anal cancer. They considered 18FDG-PET/CT for anal cancer a useful supplement in target definition for delineating smaller volume compared to CT alone and similar GTVs in comparison of MRI (23).

In our study, similarly to Drapped et al., 18FDG-PET/CT led to modify target volume of CTV N, particularly inguinal, external iliac and common iliac LN contours to include all PET-positive LN (9–11). No changes have been made to GTV T and CTV T delineation.

## Conclusion

In conclusions, 18FDG-PET/CT plays an important role in the detection of LN in patients affected by anal cancer. This could lead to a precise definition of radiotherapy target volume and dose-escalation improving tumor control. The results of our studies are in accordance with other series reported in Literature showing the usefulness of 18FDG-PET/CT in the initial staging of patients. More investigations are needed to define the role of 18FDG-PET/CT in the target volume delineation of anal cancer.

## Data Availability Statement

The raw data supporting the conclusions of this article will be made available by the authors, without undue reservation.

## Ethics Statement

Ethical review and approval was not required for the study on human participants in accordance with the local legislation and institutional requirements. The patients/participants [legal guardian/next of kin] provided written informed consent to participate in this study.

## Author Contributions

GM, CC, and MB developed the study design, collected, and interpreted patients’ data. AP and LB provided and interpreted the PET-imaging data. CC, GM, SC, and LV drafted the manuscript. CC, LV, MB, SC, AP, and GM contributed to the interpretation of the results and discussion. All authors contributed to the article and approved the submitted version.

## Funding

This work was supported by Azienda Ospedaliera Ospedali Riuniti Di Ancona.

## Conflict of Interest

The authors declare that the research was conducted in the absence of any commercial or financial relationships that could be construed as a potential conflict of interest.
